# Long Term Versus Short Term Hearing Results in Endoscopic Sandwich Myringoplasty

**DOI:** 10.22038/ijorl.2021.54372.2857

**Published:** 2021-09

**Authors:** Bikash Lal Shrestha, Ashish Dhakal, Akash Pradhan, Monika Pokharel, Pradeep Rajbhandari, Abha Kiran KC, Krishna Sunadar Shrestha

**Affiliations:** 1 *Department of Otorhinolaryngology,* *Dhulikhel Hospital, Kathmandu University Hospital, Kavre, Nepal.*

**Keywords:** Air bone gap, Endoscopy, Myringoplasty, Perichondrium, Tragal cartilage graft

## Abstract

**Introduction:**

The use of the endoscope in otological surgeries has both diagnostic and therapeutic values. It provides an excellent view in difficult nooks and corners. The use of endoscopic sandwich myringoplasty using cartilage and perichondrium has its benefit in hearing outcome and graft uptake in long-term follow-up. The main objective was to compare the long-term with short- term hearing outcomes in those who have undergone endoscopic sandwich myringoplasty with Dhulikhel hospital (D‑HOS) technique.

**Materials and Methods:**

Forty-two patients who underwent endoscopic sandwich myringoplasty with D-HOS technique using tragal cartilage perichondrium were enrolled in the study. The hearing outcome was analyzed by comparing the pre-operative findings with post-operative findings and amongst post-operative patients, long-term with short-term air bone gap (ABG) and ABG closure in speech frequencies (0.5kHz, 1kHz, 2kHz, 4kHz) were compared.

**Results:**

Amongst forty-two patients, 40 (95.2%) had graft uptake in both short-term (6.08 months) and in long-term (20 months) follow-up. The mean pre-operative ABG was 28.1±9.3dB whereas the mean short-term post-operative ABG was 14.5±7.2dB, it showed statistical significance (P=0.001). Likewise, while comparing pre-operative with long-term post-operative ABG (13.4±4.8 dB), it showed statistical significance of P=0.000. While comparing short-term with long-term post-operative ABG, it did not show any statistical significance (P=0.065).The mean closure in ABG in both short-term and long-term hearing assessment was not statistically significant (P=0.077).

**Conclusion:**

Endoscopic sandwich myringoplasty with D-HOS technique is a reliable procedure with good hearing outcome and graft uptake in both short and long-term follow-up.

## Introduction

An endoscope is nowadays commonly used in otological surgery as it provides better optics, magnification, adequate visualization and helps proper evaluation of the perforation margins, despite the narrow external auditory canal (1‑4). For the repair of perforated tympanic membrane, cartilage use is well described and was popularized by Utech in the 1950s. Good hearing results and long-term graft survival has been mentioned in different studies with the cartilage use (5-7). 

Eavey first described myringoplasty with the butterfly technique in children with the use of cartilage (8). Tos M. analyzed twenty-three cartilage tympanoplasty techniques, classifying them into 6 different categories (A to F) (9). Cartilage tympanoplasty includes different techniques like; the butterfly techniques, diced cartilage, palisade cartilage, and cartilage-perichondrium composite graft tympanoplasty (9–11). The study on endoscopic cartilage myringoplasty comparing the short term with the long‑term hearing outcome is still lacking (12-14) We did our own modification in the technique done by Rourke et al, and named it as “endoscopic sandwich myringoplasty - Dhulikhel Hospital (D‑HOS) technique”(15). In butterfly cartilage technique, splitting of the cartilage is done and groove is made in such a way that the remnant part of the tympanic membrane lies between the cartilage groove. In our technique the perichondrium supports the cartilage and the remnant perforated tympanic membrane is sandwiched between the perichondrium from the lateral end, and the cartilage with perichondrium from the medial end without elevating the tympanomeatal flap. Thus, it avoids the positional variance and strengthens the stability of the graft. The objective of our study was to compare the long-term with short‑term hearing results in patients who underwent endoscopic myringoplasty (sandwich technique) with the D‑HOS technique.

## Materials and Methods

This was a prospective and cohort study conducted from 1^st^ July 2017 to 1^st^ July 2020. Informed consent was taken from the patients before conducting the study. The inclusion criteria were: age ≥18 years, gender (both), and patient with chronic otitis media mucosal inactive type. Exclusion criteria were: Mixed or sensorineural hearing loss, revision cases, graft failure, medical or surgical conditions, or treatment that may affect the outcome.

The data collection was done in pre-operative and then in the six and twenty months post-operatively.

Pre-operatively clinical examination of ear, nose, and throat was done. Ear examination under microscope, and tuning fork tests were also performed.

*Hearing assessment*

For the hearing analysis, a pure‑tone audiogram (PTA) was performed by MAICO MA 41 (Germany) diagnostic audiometer in a sound‑treated double room set up, 7 days prior to the surgery and then after 6 months and 20 months post-operatively. The hearing was analyzed by comparing pre-operative with post-operative air bone gap (ABG) and ABG closure in four speech frequencies (0.5 KHz, 1 KHz, 2 KHz, and 4 KHz). The audiological outcome was recorded as per the guidelines given by the American Academy of Otolaryngology and Head and Neck Surgery (16).

**For the surgical procedure**

**Pre-operative workout of the patient**

Tablet Ciprofloxacin 500 mg 12 hourly was given orally to the patient a day prior to the surgery and was continued till 7th post-operative day. The surgery was performed under local anesthesia and patients were also given promethazine along with pethidine intramuscularly for sedation which was calculated as per their body weight.

**Surgical technique**

**Fig 1:**
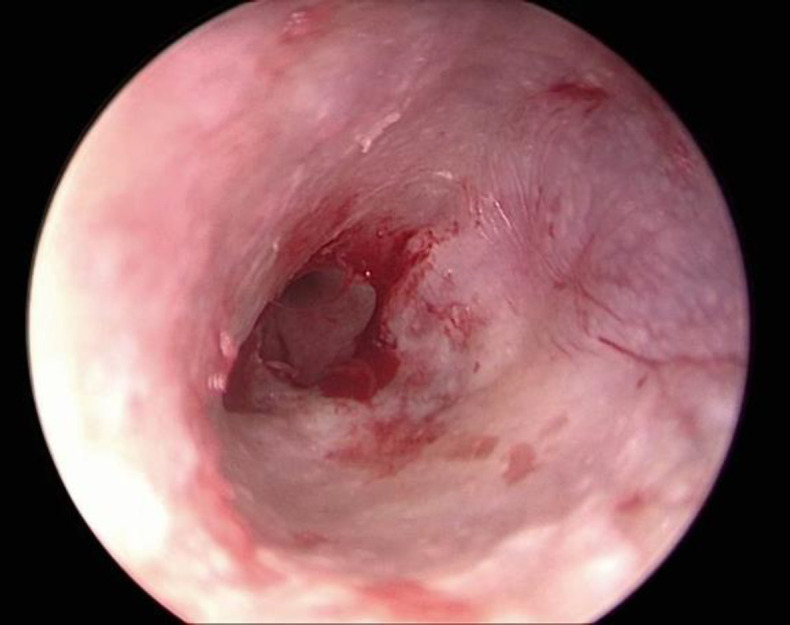
Refreshing the margin of perforation

**Fig 2:**
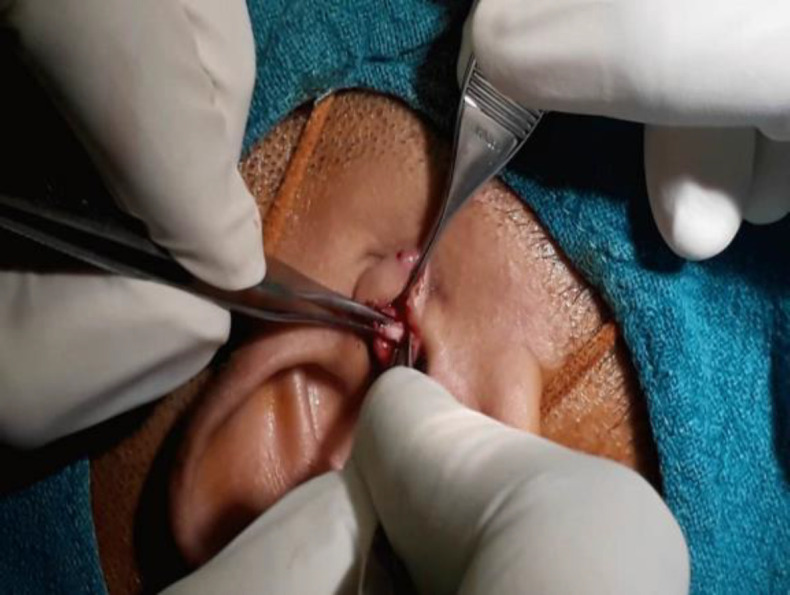
Harvesting of the tragal cartilage

Five to ten milliliter of 2% Lignocaine with 1:200,000 adrenaline was injected on the tragus and four-quadrants of the ear for ear canal block. The (Karl Storz) Hopkins II rigid endoscope both 0° and 30° with a diameter of 4mm and length of 16cm was inserted permeatally to assess the status of tympanic membrane perforation, middle ear mucosa, the ossicular chain, and the eustachian tube opening. The straight needle was used to refresh the perforation margin (Fig. 1). In conditions where the malleus handle was visible it was well skeletonized .The size of the graft was measured using a Rosen knife. The incision was given 5 millimeter medial to the tragus tip by 15 size scalpel starting from the incisura terminalis and ending at the tragal notch (Fig. 2).

The incision on the skin was given directly to the tragus cartilage. The operating surgeon dissected the cartilage along with the perichondrium both medially and laterally with the tympanoplasty scissor. During the procedure, the assistant held the tissue with non–tooth forceps as described in surgical video (*For harvesting of sandwich graft)*. Prolene suture (4/0) was used for closing the skin incision. 

**Fig 3:**
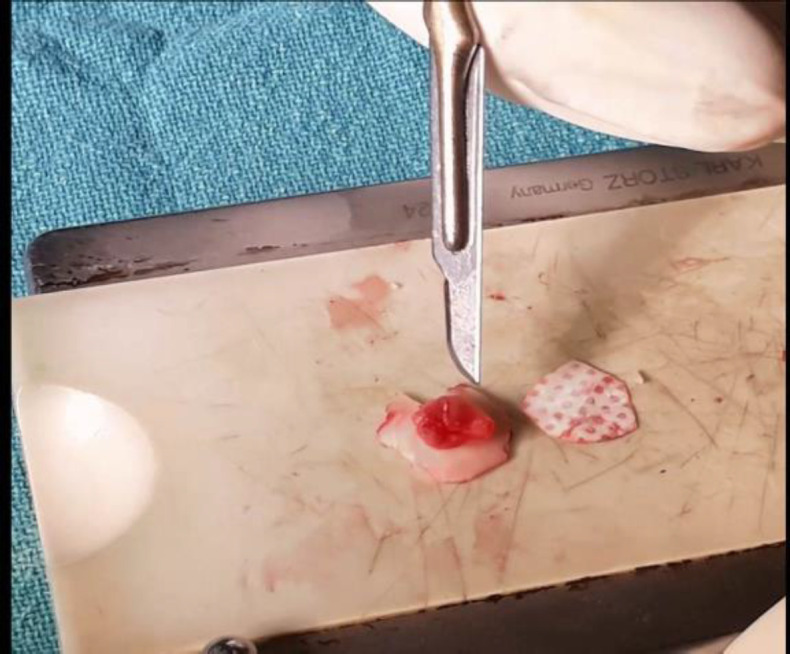
Preparing of sandwich cartilage perichondrium graft

The harvested graft was kept on the silastic block. The part of the tragal perichondrium on the central part of the cartilage was kept, elevating the remaining part of the perichondrium on the lateral side. Similarly, the tragal perichondrium on the medial side was left intact to prevent curling of the cartilage (Fig. 3).

**Fig 4:**
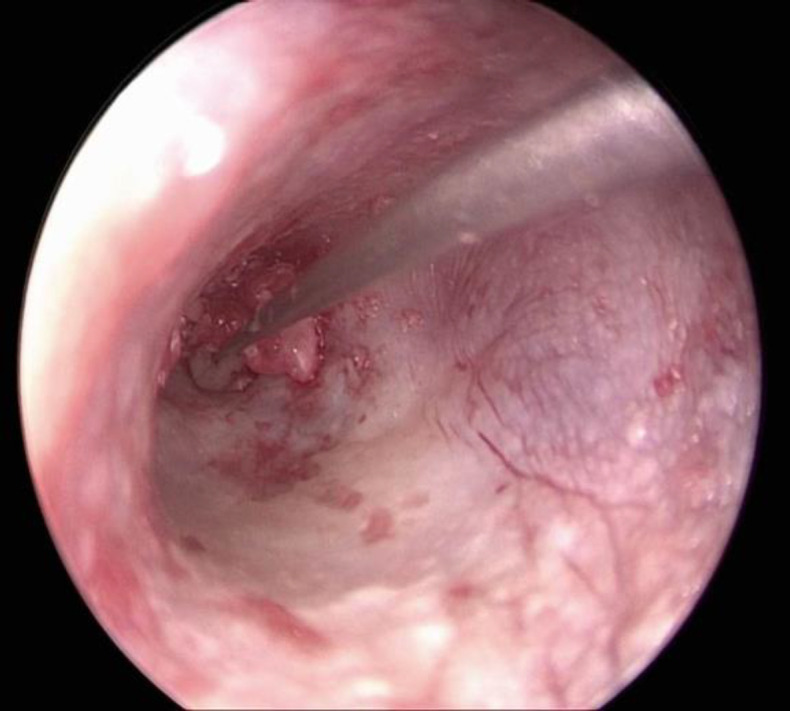
Perichondrium with cartilage lying in the middle ear and the perichondrium lies laterally on the tympanic membrane

For the graft placement, the part of the cartilage was removed to make the space for the incudostapedial joint and the malleus handle. The graft was first placed on the anterior end of the perforated tympanic membrane by holding it with the alligator forceps. Then, the remaining graft was kept in the middle ear. The elevated perichondrium was reflected to cover the lateral end of the perforated tympanic membrane thus sandwiching the tympanic membrane between the perichondrium laterally and cartilage with perichondrium medially (Fig.4).

At the end of the surgery, gelatin sponge soaked with the ciprofloxacin ear drops were placed in the ear canal and ribbon gauge medicated with soframycin were kept after that in the canal.

**Surgical video**

For harvesting of sandwich graft**: **https:// youtube/Sq95_1EJD0U Endoscopic sandwich myringoplasty D-HOS technique video: https:// youtube/MKBrKtD9 BRQ 

Post-operative follow‑up of patients

**Fig 5:**
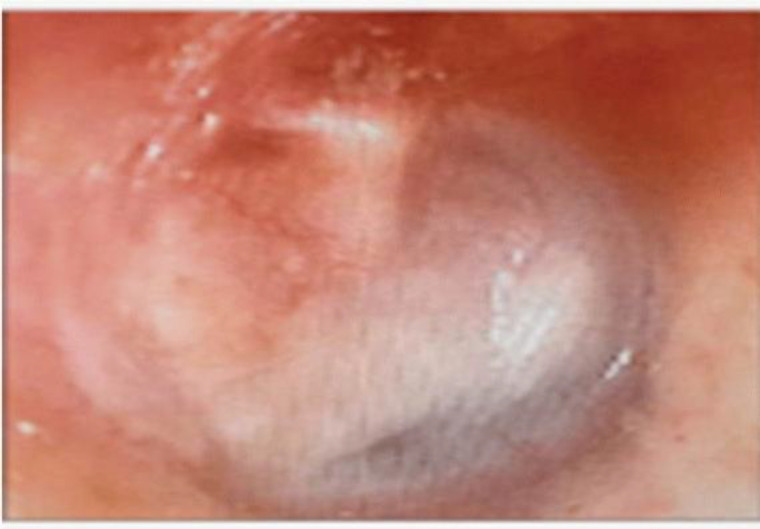
Graft uptake on 20 months of follow up

On the 7th post-operative day, the ribbon gauge and the gelatin sponge were removed from the external auditory canal. The stitches on the tragal area were also removed. The patient was given the chloramphenicol and dexamethasone ear drops for the next 6 weeks. Follow-up of the patient was done at 6 months and 20 months post-operatively to see for graft uptake and hearing outcome (Fig.5).

**Statistical analysis**

For the analysis of statistical data, ENT statistics software (Otology Module) Client Version: 4.0.0.14, Pro edition, DB version (normal model): INNOFORCE creative solutions, ENT statistics DBIII-3. 0-492 from Liechtenstein, 2019, was used. For analyzing the data, the student’s t‑test was used and the p-value of < 0.05 was set for significance level.

**Ethical clearance**

The ethical clearance was taken from the Review Committee of Kathmandu University School of Medical Sciences, Dhulikhel (IRC-KUSMS: 34/18). The study was performed as per the Helsinki Declaration principle.

## Results

A total of 42 patients were enrolled in the study. Among them, two cases had perforated tympanic membrane after surgery due to infection, and hence, only 40 patients were included. Among them, 18 were males and 22 were females. The age distribution ranged from 18 to 55 years with 25.1 ± 1.09 years. The graft uptake rate was 95.2% in both the short-term (6.08 months) and in long-term (20 months) follow-up. We did not observe other complications like blunting of anterior angle, lateralization of graft, myringitis or cholesteatoma formation, sensorineural hearing loss, vertigo, tinnitus, or facial palsy during 20 months follow-up period. The surgical time was 30±13 minutes from skin incision to graft placement. The pre and post‑operative hearing level (HL) is shown in Table 1, with a statistically significant improvement when comparing short-term with long-term HL in the post-operative period.

**Table 1:** Showing the demographic data, graft uptake and hearing results in short versus long-term outcome. (n=40)

**Number of cases **	**42 (2 excluded – graft failure)**	
Gender		
Male	18	
Female	22	
Mean age	25±1.09 years	
Mean follow up (months)		
Short-term	6.8 months	
Long-term	20 months	
Surgical procedure	Endoscopic sandwich myringoplasty (D-HOS Technique)	
Right:Left side	22:18	
Surgical time	30±13minutes	
Graft success rate		
Short-term	95.2%	
Long-term	95.2%	
Mean pre-ope HL (dB)	39.6±16.3	
Mean post-ope HL (dB)		
Short-term	20.4±9.7	P = 0.041
Long-term	17.6±7.7	
Mean pre-ope ABG (dB)	28.1±9.3	
Mean post-ope ABG (dB)		
Short-term	14.5±7.2	p = 0.065
Long-term	13.4±4.8	
Mean ABG closure		
Short-term	13.6±2.1	P=0.077
Long-term	14.7±4.5	

**Fig 6:**
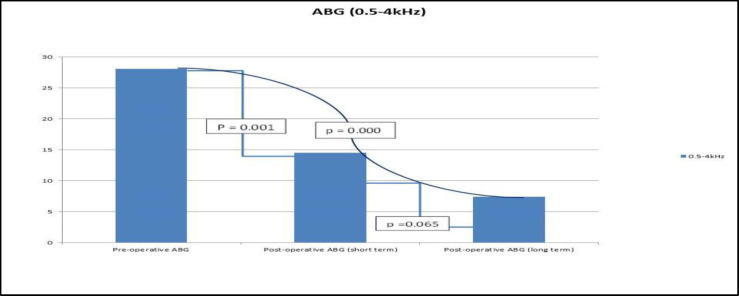
Showing the comparison of ABG [Pre versus post-operative (short term and long term)]

As shown in table 1 and figure 6, comparison of ABG, pre with short-term post-operative, and pre with long-term post-operative showed statistically significant results whereas comparing short-term with long-term ABG was not statistically significant. The comparison of short-term ABG closure with the long-term was not statistically significant as shown in table 1.

**Fig 7:**
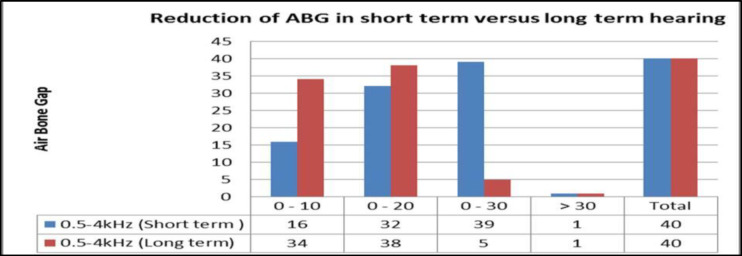
*Showing the ABG reduction between short and long term hearing (n=40)*

**Fig 8:**
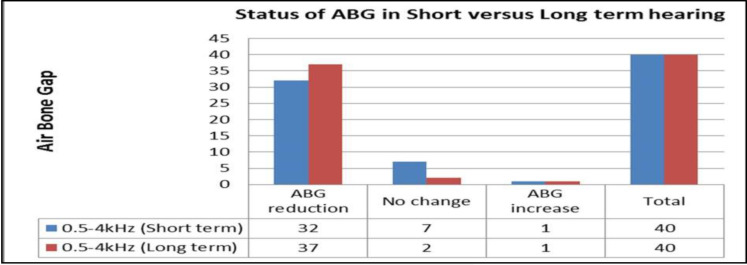
*Showing the status of ABG in short and long term hearing (n=40)*

Figure 7 shows the ABG reduction within 20dB in 80% cases in the short-term and 95% in the long-term hearing outcome. The total ABG reduction was 80% in short-term and 92.5% in the long-term hearing assessment as shown in figure 8.

## Discussion

This study focuses on the surgical outcome and audiological results following endoscopic sandwich myringoplasty with D‑HOS technique in 40 patients. In our technique, we modified in a way, that the perforated tympanic membrane’s lateral part was covered by the elevated part of the perichondrium and the intact cartilage with perichondrium lied medial to the perforation, thus sandwiching the tympanic membrane between cartilage with perichondrium medially and only perichondrium laterally. This particular technique gave stability to the graft and also avoided variation in position of the graft. The tympanic membrane covered laterally by the perichondrium worked as a scaffold for the outer migration of squamous epithelium (17). We observed that the use of endoscope aided excellent visualization of the anterior end of perforation and 360° view of the perforated tympanic membrane. The graft could be easily kept with the help of endoscope without taking a long time in our D‑HOS technique, which took only 30±13minutes and it did not require tympanomeatal flap elevation (18,19). While raising the tympanomeatal flap, there are increased incidence of bleeding in the external auditory meatus and this created fogging of the endoscope. This also increased the surgery time (20,21).Studies showed that limited or not raising the tympanomeatal flap helped in early recovery of the wound and continuous perfusion lead to successful graft uptake (21,22). We have used tragal cartilage and perichondrium as the graft material because the different experimental and clinical studies revealed that the cartilage survived for a long time, with minimal resorption time and good hearing outcome (7,23-26). Similarly, keeping the perichondrium intact on either side had better survival due to good metabolism and tolerated the strong enzymatic reaction than cartilage without perichondrium (27). There were different methods of cartilage tympanoplasty for the graft procedure mentioned in the literature (28,29). The technique of butterfly cartilage was described by Eavey for small‑to‑medium sized perforation (8). Later on, Rourke et al. and Ghanem et al. modified this butterfly technique to repair the large perforation (15,30). We performed the technique with our modification, keeping both sides of the perichondrium intact and named it as “The sandwich myringoplasty D‑HOS technique” because the margin of the perforation was sandwiched on one side by the perichondrium only and another side by the cartilage and the perichondrium. 

The advantages of our technique are:

It is more comfortable technique as there is no need to raise the tympanomeatal flap and less time consuming. The perforated tympanic membrane was sandwiched on either side maintaining the graft position without support, either from the outer canal or the middle ear and there was very minimal oozing (27).

There are certain disadvantages we noticed:

Learning curve is more, it requires the exact measurement of the tympanic membrane perforation for preparing the graft, lots of practice is needed in precisely raising the perichondrium flap at lateral side to make island at the center area of the cartilage. Learning curve for using the endoscope is more as it is a single-handed technique; and finally, the theoretical disadvantages of using the cartilage is that it causes opaque appearance of tympanic membrane and can hide the disease in the middle ear cavity (30).The possibility of an outer layer of the tympanic membrane migrating its way to the middle ear from the medial side of the perichondrial layer is stated in theory, but in our study we did not find any cholesteatoma which was iatrogenically induced during 18-24 months follow‑up period. Similarly, previous studies done by various authors also did not mention the butterfly tympanoplasty technique leading to cholesteatoma (8,27,31). The reason is the tympanic membrane being covered laterally by the perichondrium worked as a scaffold for the outer migration of squamous epithelium (17). Hence, we can tell that it is a credible method in this regard. However, long-term follow-up of 5–10 years will be required to know the development of cholesteatoma and we are following these patients for long term complications. The graft success rate in our study is comparable to the different studies in the literature. The graft success rate was 95.2% in both short and long-term follow up in our study, which is comparable to other studies which showed a success rate from 73% to 97% as shown in table 2 (17,32-46).

**Table 2:** Showing the graft uptake rate in literatures

**Author**	**N**	**Graft material used**	**Graft uptake rate (%)**	**Surgical method**
Lau^17^	67	Cartilage	97	Endoscopic double layer
Raj and Meher^32^	20	Cartilage	90	Endoscopic transcanal
Zhang et al.^33^	43	Cartilage	95	Endoscopic modified sandwich technique
Ayache^34^	30	Cartilage	96	Endoscopic underlay
Celik et al.^35^	32	Cartilage	87.5	Endoscopic push through underlay
Omran^36^	30	Cartilage	73.3	Endoscopic bivalve inlay
Özgür et al.^37^	45	Cartilage	97.8	Endoscopic butterfly
Mokbel et al.^38^	80 (40 each in endoscopic microscopic group)	Cartilage	100 in endoscopic and 90 in microscopic	Endoscopic transcanal and microscopic transcanal
Garcia et al.^39^	22	Cartilage	86.4	Endoscopic inlay
Kaya et al.^40^	93	Cartilage	94.6	Butterfly cartilage
Karabulut et al.^41^	56	Cartilage	98.2	Endoscopic butterfly inlay
Nemade et al.^42^	46	Cartilage	95.8	underlay
Bedri et al.^43^	390	Cartilage	90	Double layer
Chhapola and Matta^44^	61	Cartilage	98.36	composite
Parelkar K et al^45^	39	Cartilage	78	Endoscopic shield
Daneshi A et al^46^	75	Cartilage	97.3	Endoscopic transcanal
				

The hearing outcome following myringoplasty is mainly affected by the conditions of the ossicular chain, residual perforation of the tympanic membrane, graft uptake, and lastly the medialization or the lateralization of the intact graft (18). While analyzing the audiological results, different studies reported the post-operative ABG decreased in butterfly cartilage tympanoplasty. The study performed by Özgür et al. on endoscopic butterfly inlay myringoplasty showed that the mean ABG was 9.4 dB on the 6^th^ post-operative month in adult group (37). The study performed by Kaya et al. showed that the mean air conduction in butterfly cartilage tympanoplasty was better in 24 months follow up as compared to 6 months follow up (40). The study done by Karabulut et al. revealed that the mean pre-operative ABG was 24.2 ± 3.8 dB, whereas the mean post-operative ABG on the 12^th ^and the 24^th^ month, was 17.1 ± 3.5 dB and 12.4 ± 3.1 dB respectively (41). The study performed by Lou showed that the mean pre-operative ABG was 23.26 ± 8.34 dB. The mean post-operative ABG on 6 months follow up was 11.35 ± 3.27 dB, and on 12 months follow up was 9.61 ± 2.54 dB which was statistically significant (17). Our study showed that the mean pre-operative ABG was 28.1±9.3 dB and the mean post-operative ABG on 6 months follow‑up was 14.5±7.2dB which showed statistically significant results. Similarly, the mean post-operative ABG on the 20 months follow‑up was 13.4±4.8dB with statistically significant results when compared with the pre-operative ABG results. But, it was statistically not significant when comparing short-term with the long-term results. Hence, our audiological results are comparable with the above-mentioned studies and with other different studies which showed significantly improved post-operative ABG (32-46). Our study showed that the mean ABG closure was13.6±2.1dB in short-term hearing evaluation whereas it was 14.7±4.5dB in long-term hearing evaluation which is also comparable with different studies (14,32-39,43,45,46). Similarly, the ABG reduction was 80% in short-term hearing evaluation and 92.5% in long-term hearing evaluation, whereas ABG reduction was within 20dB in 80% of cases in short-term and 95% of cases in long-term hearing evaluation which is similar to the study done by Chhapola et al (44).In our study, the mean time for post-operative short-term follow-up for hearing analysis was 6.8 months (range, 5.01 to 8.30 months) whereas the follow-up for long-term hearing analysis was 20 months (range, 18.40 to 36.11 months). The long-term follow-up of our study gave us the idea about the dynamic changes in hearing in our D-HOS technique. The good hearing outcome in our study in both the short-term and in the long-term period could be because of the modification we did. In our technique only the perichondrium part lies at the malleus handle and the incudostapedial joint wherever visible, this could be the reason for better hearing due to the better conduction of sound.

The main limitations of our study are:

1. Sample size.
2. Single institutional study.

## Conclusion

Endoscopic sandwich myringoplasty (D-HOS technique) has both good short-term as well as long-term hearing results. Apart from that, it has an excellent graft uptake rate. It is less time consuming and has very good cosmesis. Hence, we recommend to perform this technique.
